# Modeling of the growth of GaAs–AlGaAs core–shell nanowires

**DOI:** 10.3762/bjnano.8.54

**Published:** 2017-02-24

**Authors:** Qian Zhang, Peter W Voorhees, Stephen H Davis

**Affiliations:** 1 Department of Engineering Sciences and Applied Mathematics, Northwestern University, 2145 Sheridan Road, Evanston, Illinois 60208-3125, USA; 2 Department of Materials Science and Engineering, Northwestern University, 2225 Campus Drive, Evanston, Illinois 60208-3030, USA

**Keywords:** core–shell nanowires, heterostructures, mechanisms, quantum dots

## Abstract

Heterostructured GaAs–AlGaAs core–shell nanowires with have attracted much attention because of their significant advantages and great potential for creating high performance nanophotonics and nanoelectronics. The spontaneous formation of Al-rich stripes along certain crystallographic directions and quantum dots near the apexes of the shell are observed in AlGaAs shells. Controlling the formation of these core–shell heterostructures remains challenging. A two-dimensional model valid on the wire cross section, that accounts for capillarity in the faceted surface limit and deposition has been developed for the evolution of the shell morphology and concentration in Al*_x_*Ga_1−_*_x_*As alloys. The model includes a completely faceted shell–vapor interface. The objective is to understand the mechanisms of the formation of the radial heterostructures (Al-rich stripes and Al-poor quantum dots) in the nanowire shell. There are two issues that need to be understood. One is the mechanism responsible for the morphological evolution of the shells. Analysis and simulation results suggest that deposition introduces facets not present on the equilibrium Wulff shapes. A balance between diffusion and deposition yields the small facets with sizes varying slowly over time, which yield stripe structures, whereas deposition-dominated growth can lead to quantum-dot structures observed in experiments. There is no self-limiting facet size in this case. The other issue is the mechanism responsible for the segregation of Al atoms in the shells. It is found that the mobility difference of the atoms on the {112} and {110} facets together determine the non-uniform concentration of the atoms in the shell. In particular, even though the mobility of Al on {110} facets is smaller than that of Ga, Al-rich stripes are predicted to form along the {112} facets when the difference of the mobilities of Al and Ga atoms is sufficiently large on {112} facets. As the size of the shell increases, deposition becomes more important. The Al-poor dots are obtained at the apices of {112} facets, if the attachment rate of Al atoms is smaller there.

## Findings

**Figure 1:**
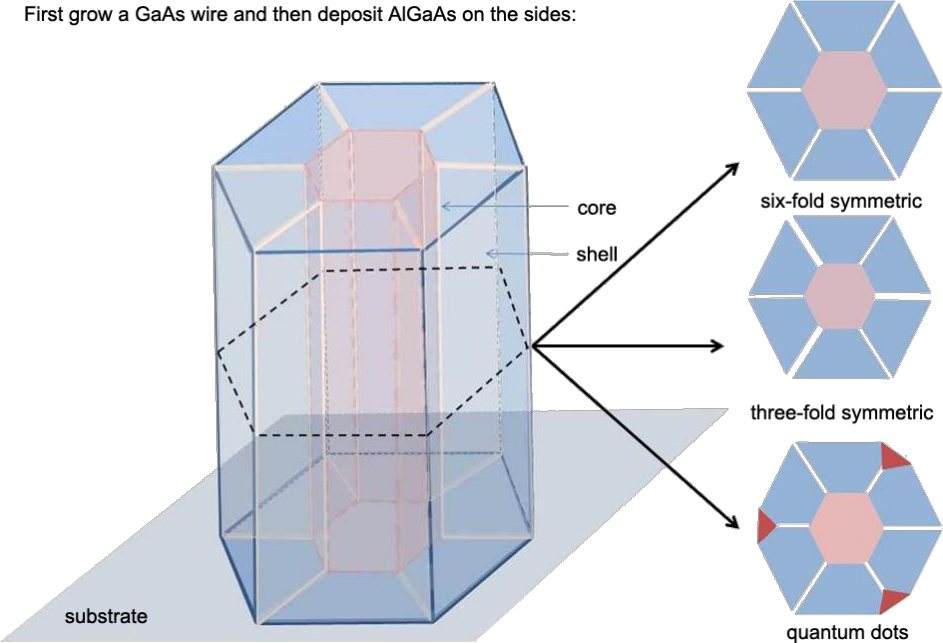
Schematic of a core–shell nanowire (left) with possible configurations of the cross section (right) observed in experiments. These are the six-fold symmetry configuration observed in [3] with Al-rich {112} facets along the corners of the hexagonal core (top right); the three-fold symmetry configuration observed in [4] (middle right) and the Al-poor quantum dots (red) at the apices adjunct with the Al-rich stripes observed in [2] (bottom right).

Core–shell nanowires with heterostructures hold great promise in photonic and electronic applications because of their high sensitivity to electronic and magnetic fields. However, controlling the formation of these heterostructures remains a challenge because they are typically embedded in 3D matrices. One of the potential solutions to this problem is to create heterostructures near the edges of the nanowires. Classical approaches to create this kind of heterostructure are to deposit materials with lattice parameters different from that of the wires, such as growing a Ge shell on a Si nanowire [1], so that the quantum dots form as a result of stresses generated by the lattice parameter mismatch. Recently, GaAs has been used as substrates to deposit AlGaAs on their sides. Heiss et al. [2] observed that the deposition of the AlGaAs layer leads to Al-poor quantum dots located within the fork-like Al-rich stripes along the 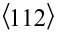 directions (Figure 1).

Yet, there is a very small elastic mismatch between AlGaAs shell and GaAs core. So, the question is, how do these heterostructures form in this experiment? Moreover, many other experiments also observe radial heterostructures with the same materials [3]. Jiang et al. [5] also observed Al-rich stripes along the 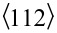 directions. Zhang et al. [4] obtained a three-fold symmetry structure with Al-rich stripes (Figure 1). Thus, an understanding of the mechanisms leading to these heterostructures could be very helpful in controlling the formation of these core–shell nanowires.

There are two issues that need to be understood. One is the mechanism responsible for the morphological evolution of the shells. The surface energy density on the {112} facets is at least three times larger than that on the {110} facets. Energy minimization thus tells us that there are no {112} facets in the equilibrium state. Thus, the question is, how do the {112} facets, which are not present on the Wulff shape, arise in the configuration of the shell of nanowires? The other issue is the mechanism responsible for the segregation of Al atoms in the shells. More specifically, under certain deposition conditions, Al atoms move slower than Ga atoms along the {110} facets [6]. If the atoms move from {110} to {112} facets, then the Al atoms should be left behind on the {110} facets rather than accumulate in the 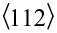 directions. Thus, the question is how the Al-rich stripes could form along {112} facets when the atoms move from {110} facets to {112} facets and the mobility of Al is lower than that of Ga on {110} facets [6]. Following this question is that of how the Al-poor dots form directly following the Al-rich stripes.

**Figure 2:**
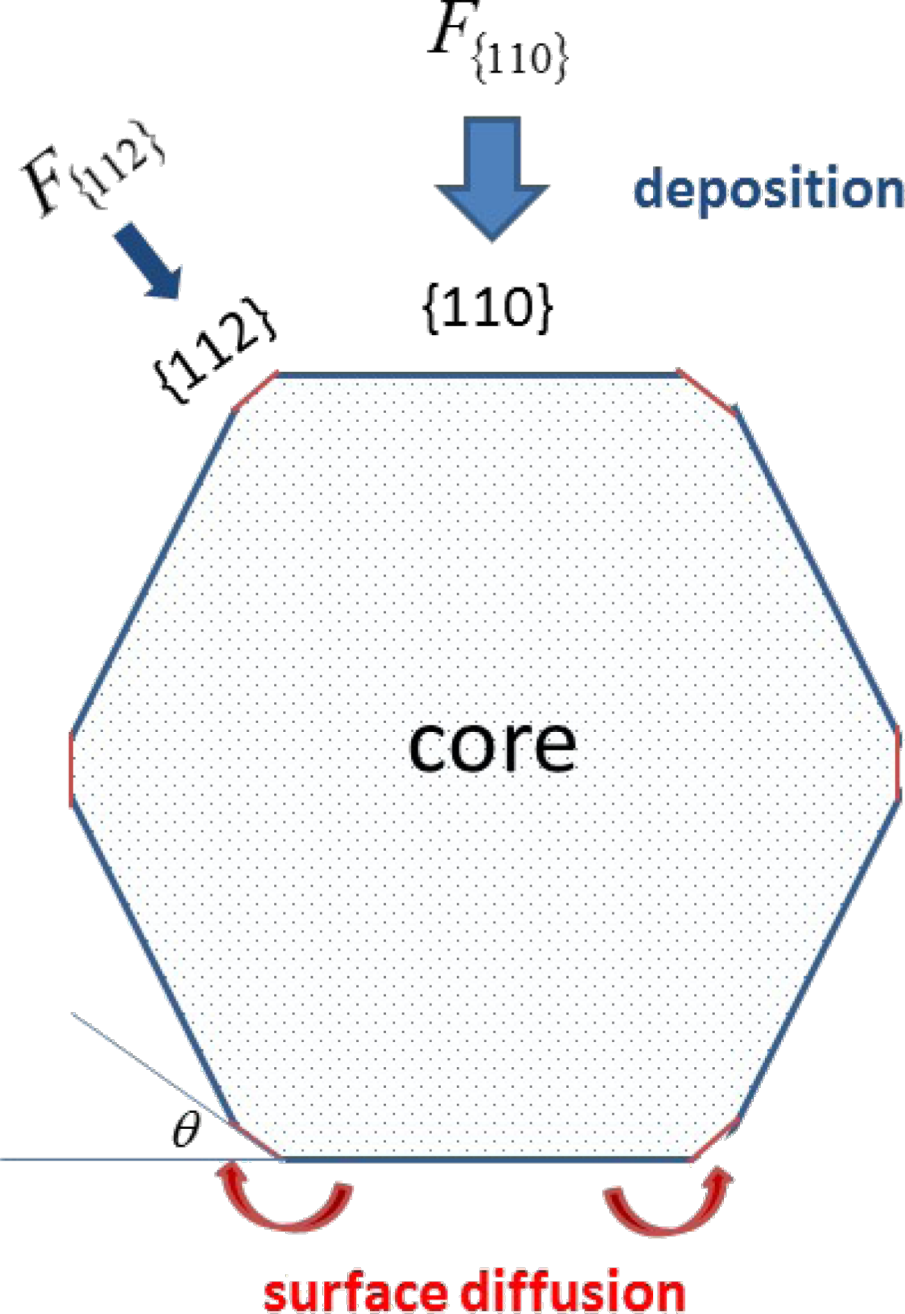
Schematic of cross section of a core of a nanowire that is a six-fold symmetric structure with {112} facets along the corners of the hexagonal core. The lengths of the facets are *l*_{112}_ and *l*_{110}_, respectively. In this structure, the angle θ between a {110} facet and its neighboring {112} facet is π/6.

These problems are solved in [7] and [8], respectively. For simplicity, at first a pure material is deposited around the hexagonal core to investigate the mechanisms governing the morphological evolution. In [7], we proposed a mathematical model for the deposition of pure materials onto a faceted nanowire. In the model, the growth of each facet is due to the deposition of atoms from the vapor and surface–atom diffusion from its neighbouring facets (Figure 2).

It is suggested in experiments that bulk diffusion is negligible relative to surface diffusion for typical temperatures and length scales. Moreover, the motion of the atoms along the surface due to surface diffusion is driven by the difference of the chemical potentials between neighbouring facets. The average chemical potential on the *i*-th facet is calculated by

[1]
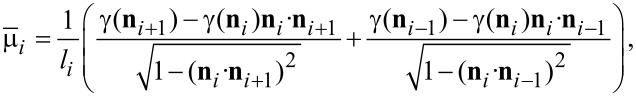


where **n***_i_* is the unit normal vector of the *i*-th facet and γ(**n***_i_*) is the surface energy density on the *i*-th facet. Thus, if there is deposition, the mass conservation around the surface gives the following equations:

[2]
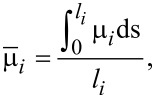


[3]
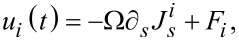


[4]
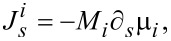


where *u_i_* is the normal velocity of the *i*-th facet, 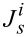 is the surface diffusion flux along *i*-th facet and μ*_i_* is the chemical potential on the *i*-th facet, Ω is the atomic volume and *F_i_* is the total deposition rate of the material in the outer normal direction of the *i*-th facet, *M_i_* is the mobility of the atoms on the *i*-th facet, and *l_i_* is the length of the *i*-th facet. Equation 2–Equation 4 together with the continuity conditions of surface diffusion flux and chemical potential at the corners give a complete equation system of the evolution of the configuration of a crystal. Thus, facet lengths evolve as

[5]
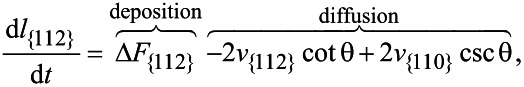


[6]
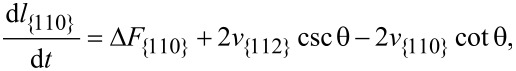


where Δ*F*_{112}_ = −2*F*_{112}_cotθ + *2F*_{112}_cscθ, Δ*F*_{110}_ = *2F*_{112}_cscθ − *2F*_{110}_cotθ (θ is shown in Figure 2 and θ = π/6 in this six-fold symmetric configuration). Here, *v*_{112}_ and *v*_{110}_ are the normal velocities of the corresponding facets due to diffusion of the surface atoms. This fully faceted model without deposition flux was first proposed by Carter and co-workers [9]. The derivation for the continuum model can be found in [7,10–12]. Different from the focus of [9], in [7], we did a systematical theoretical analysis and numerical simulations for the case shown in Figure 2.

**Figure 3:**
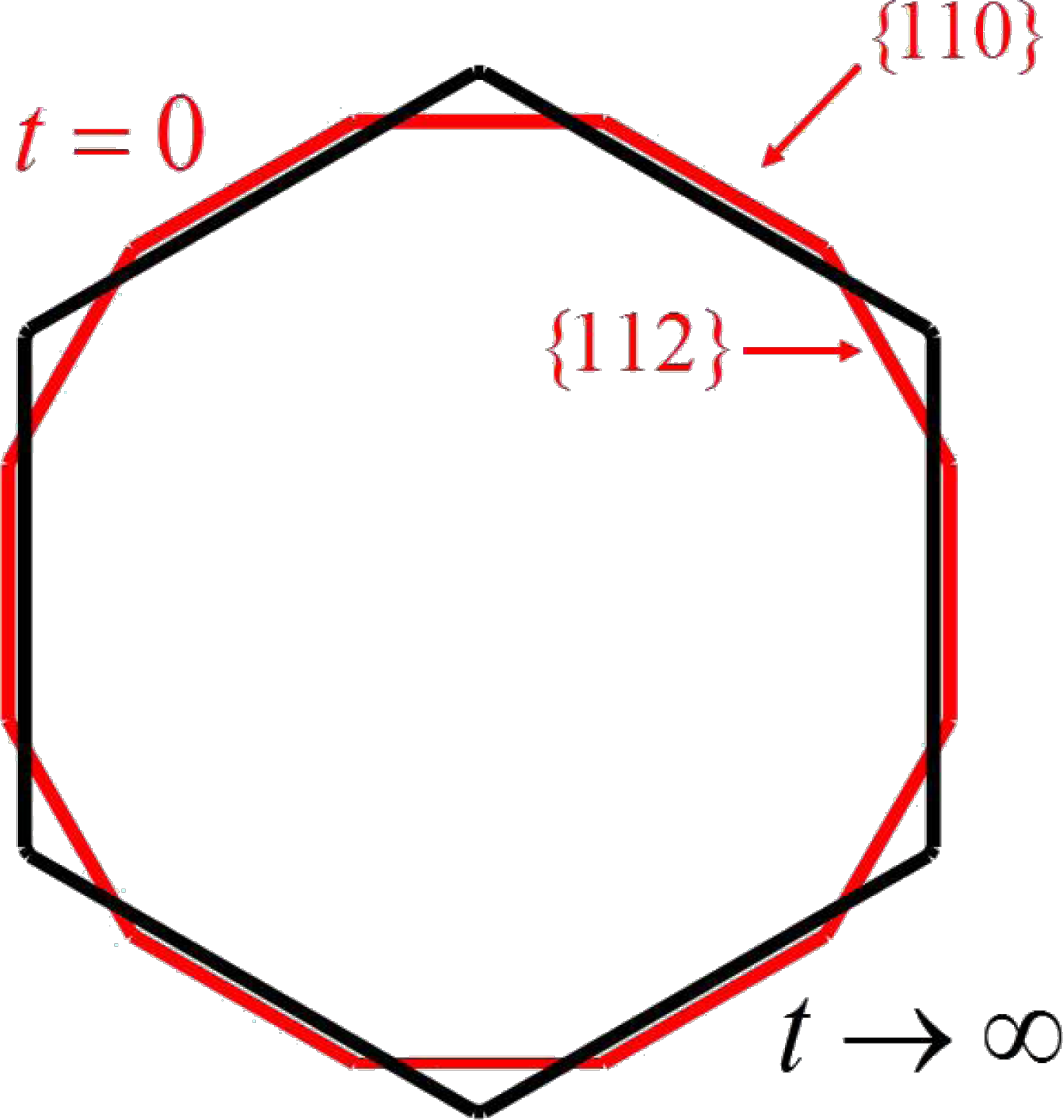
Evolution of a dodecagonal crystal (bounded by red lines) to a hexagonal crystal (bounded by black lines) due to surface diffusion.

Here, in order to determine the influence of diffusion and deposition on the morphological evolution of the shell, consider first a surface evolution due only to surface diffusion, i.e., without deposition. Figure 3 shows the evolution of a dodecagonal crystal with initially *l*_{112}_ = *l*_{110}_ (red lines) to a hexagonal crystal due to surface diffusion. Under the condition γ_{112}_ = 3γ_{110}_, Equation 1 implies that 
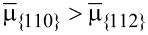
. Thus, surface diffusion tends to move the atoms from {110} to {112} facets and eventually the {112} facets disappear (black line). In addition, through this numerical example, it is seen that the dynamic simulation result yields the equilibrium Wulff shape.

**Figure 4:**
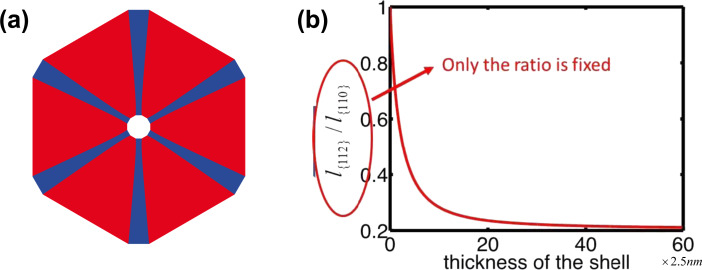
(a) Evolution of a dodecagonal shell to its kinetic Wulff shape due to surface diffusion and deposition. (red: six {110} facets; blue: six {112} facets.) (b) Evolution of the ratio between the lengths of the facets shown in (a).

Now, consider deposition in the process of growth of the shell with the same conditions as the last numerical simulation, i.e., *F*_{112}_ = 1.1*F*_{110}_ with *F*_{110}_ being large enough. These deposition rates can be different on facets of different orientations due to different sticking coefficients or exchange rates between the surface and bulk. Instead of getting a hexagonal shape, a dodecagonal shell is obtained (Figure 4a). Moreover, as the thickness of the shell gets large, the ratio between the lengths of the facets approaches a non-zero constant, i.e., *l*_{112}_/*l*_{110}_ ≈ Δ*F*_{112}_/Δ*F*{110} = 0.202 (Figure 4b). This is because diffusion becomes negligible compared with deposition as the size of the shell gets large (see Equation 6). Moreover, when the thickness of the shell is large enough, this ratio does not change, even though the size of the facets continue to grow; this is a self-similar shape, the “kinetic Wulff shape” because it is obtained when kinetic effects dominate. Thus, it is shown that deposition allows for the possibility of {112} facets that are not present on the equilibrium Wulff shape.

**Figure 5:**
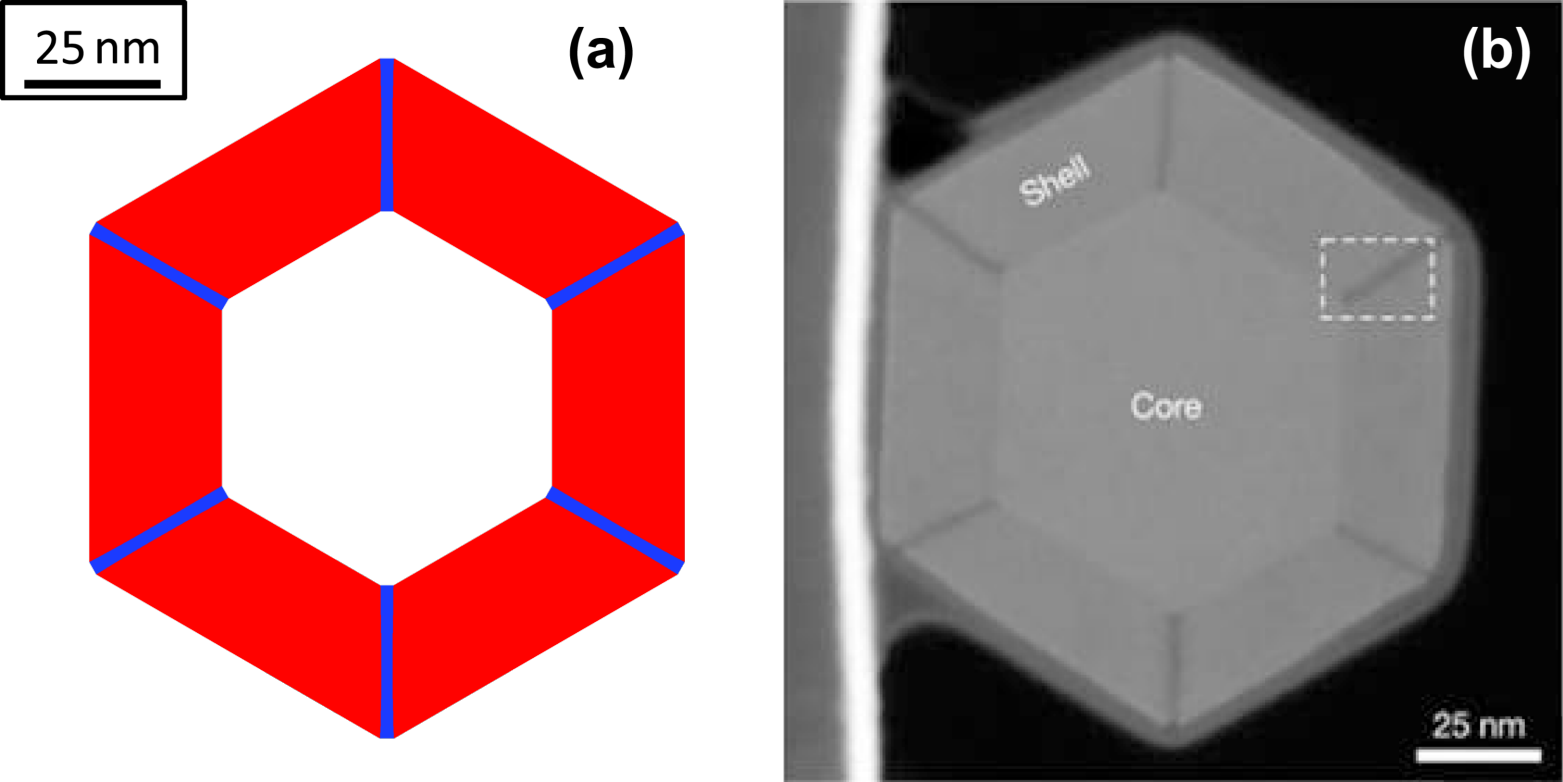
(a) Cross section of the core–shell nanowire with six {112} facets (blue color) along the corners of six {110} facets (red color) in the numerical simulation; the stripe structure along the 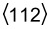 directions within 25 nm of thickness of the shell. (b) The experimental result showing Al-rich stripes along the 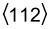 directions (dark lines) obtained in [3].

**Figure 6:**
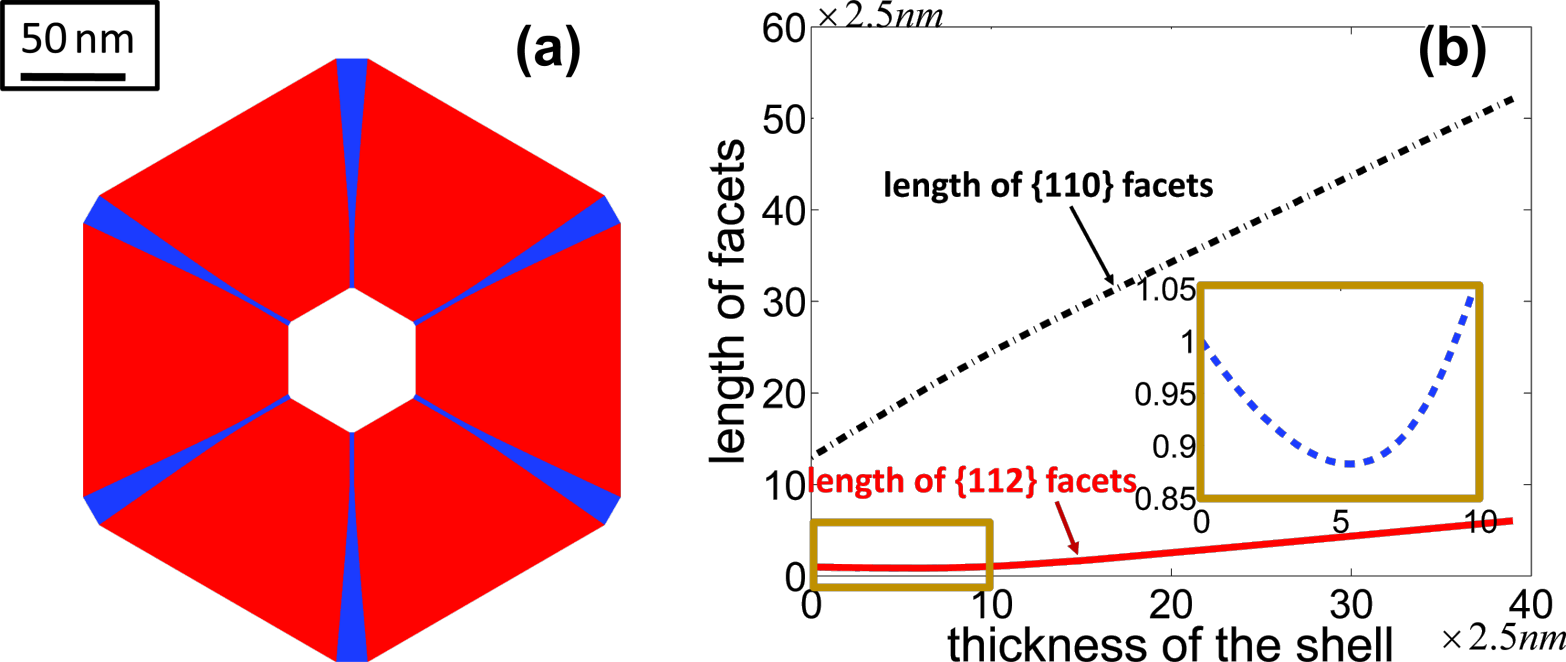
(a) Cross section of the core–shell nanowire with six {112} facets (blue color) along the corners of six {110} facets (red color) in the numerical simulation when the shell of the nanowire is large enough. (b) Evolution of the length of the facets with respect to the thickness of the shell.

According to the discussions above, surface diffusion tends to remove the {112} facets whereas deposition tends to preserve them. Figure 5a shows the balance between the surface diffusion and deposition giving a slowly varying facet size at early times. The numerical simulation is compared with the experiment result in [3] (Figure 5b) giving almost quantitative agreement. When a certain thickness of the shell is reached, the size of the {112} facets increases rapidly (Figure 6). As shown in the inset in Figure 6b, the length of {112} facets evolves with the increasing of the thickness of the shell, even though the rate of change is much smaller than that of {110} facets at early time. This implies that the length of the {112} facets is never constant over time.

This conclusion departs from the traditional understanding on the formation of the Al-rich stripes along the 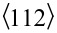 directions. In [2–3,13], the claim is that the Al-rich stripes form because the length of the {112} facets reaches its “self-limiting” size and does not change further upon growth of the shell. Such self-limiting facets are absent in our results. (In order to see that such a self-limiting facet size does not exist, we argue by contradiction. Suppose there was a self-limiting facet size for the {112} facets, as the size of the shell of the nanowire increases, the length of {110} facets must then increase. Thus, the ratio of the length of the {112} facets to the length of the {110} facets cannot be a constant. This contradicts the attainment a self-similar shape, i.e., the kinetic Wulff shape in which the ratio of the length of {112} facets to the length of {110} facets is a constant, when the size of the nanowire is large enough.) Rigorous proof of the non-existence of a self-limiting facet size can be found in [7].

**Figure 7:**
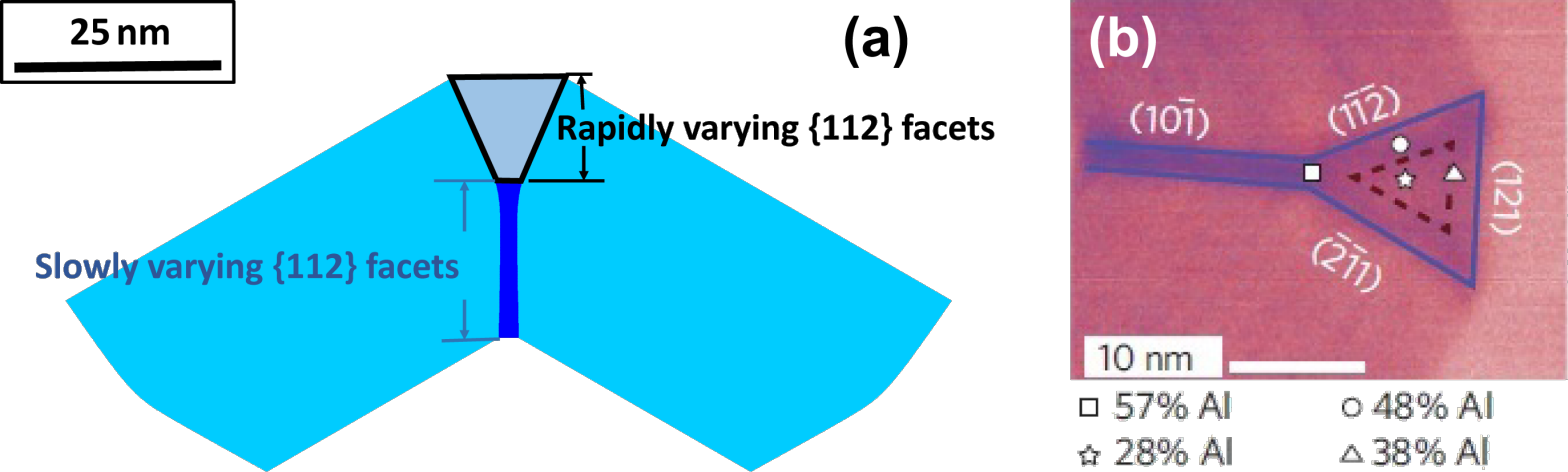
(a) This is partial view of a cross section of the shell of core–shell nanowire with one {112} facet along the corner of two neighboring {110} facets. (b) Detail of a Al-poor quantum dot located within the fork-like Al-rich stripes obtained in [2].

Given that there is no self-limiting size for any facet, another set of deposition rates can yield the dot configuration that is formed spontaneously by a transient growth (Figure 7b). The configuration obtained in the numerical simulation is very close to that seen experimentally (Figure 7a).

Besides the dot configuration shown in Figure 7b, Heiss et al. [2] also observed the segregation of Al in the shell of the nanowire (see the Al concentration shown in Figure 7b). It is obvious that a model assuming pure materials cannot address the mechanisms leading to the non-homogeneous distribution of Al in the shell. A two-dimensional fully faceted model [8] was developed for the growth of the shell with an A*_x_*B_1−_*_x_* (Al*_x_*Ga_1−_*_x_*As) alloy on a hexagonal core. Surface morphology and composition on the *i*-th facet evolve as

[7]
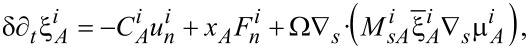


[8]
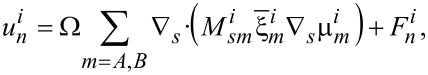


where δ is the ratio of the density of lattice sites on the surface to that in the bulk. For each component *m*, ξ*_m_* is the local composition fraction of atoms *m* of the surface layer and


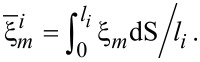


Moreover, ξ*_A_* + ξ*_B_* = 1. 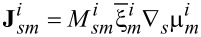 is the surface-diffusion flux of component *m*. μ*_m_* is the chemical potential at the surface. *M_sm_* is the surface diffusivity of atoms *m*. *F_n_* is the total deposition rate of atoms and is positive for growth, and *x_m_* is the fraction of component *m* on the surface due to the deposition flux. *C_m_* is the local composition of the “bulk” material immediately beneath the surface layer. This model suggests that the changing rate of the concentration of A atoms is due to the surface diffusion of the atoms along the surface, the physical deposition of the atoms from the deposition fluxes, and the motion of the surface, which leads to the atoms leaving or entering the surface from below. Similar to the pure-material model, the chemical potentials can be related to the surface energy density (γ) and bulk energy density (*g*_α_) in its average sense, as follows:

[9]
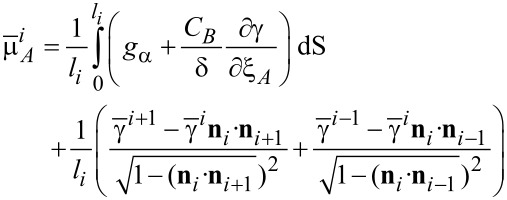


[10]
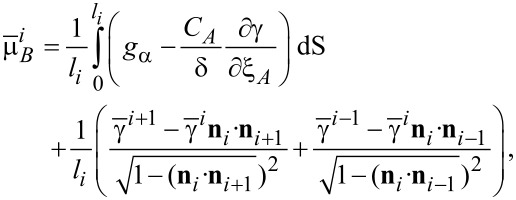


where


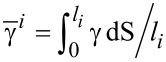


is the average surface energy density of the *i*-th facet. In addition, the advancing surface leaves behind material with composition *C_A_* determined by the equilibrium with the surface layer 1/δ. Equation 7–Equation 10 cooperating with the continuity conditions at the joint points of two neighboring facets for the surface diffusion fluxes, the chemical potentials at the surface, and the surface concentrations give a complete system for the evolution of morphology and surface concentration. A derivation of the model on faceted surfaces with anisotropic surface energy density and numerical simulation details can be found in [8]. Similar models on smooth surfaces with isotropic surface energy density were proposed in [14–15].

The mobility of Ga atoms on the {110} facets is difficult to measure. However, the measurement in [6] gives a diffusion of Ga that is ten times larger than that of Al atoms, i.e., 
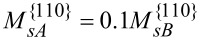
. The diffusivities of Al and Ga are likely functions of surface reconstructions and thus can be functions of the deposition conditions. For simplicity, take


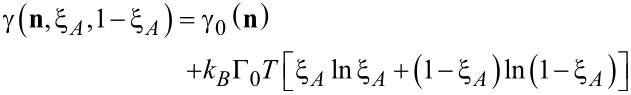


and


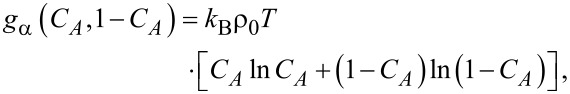


where γ_0_(**n**) is the surface energy density for pure atoms A or atoms B, *k*_B_ is Boltzmann’s constant, *T* is the absolute temperature, Γ_0_ and ρ_0_ are the lattice sites densities on the surface and in the bulk, respectively. In the case considered here *u_n_* *>* 0, the advancing surface leaves behind material with composition *C_A_* determined by the equilibrium with the surface layer, (1/δ)∂γ/∂ξ*_A_* = *dg*_α_/*dC_A_*[8,14,16–17]. In experiments, 
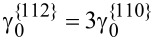
 and γ_0_ ≈ 1 J/m^2^, Γ_0_ ≈ 10^18^/m^2^, ρ_0_ ≈ 10^28^/m^3^ and *T* ≈ 1000 K. Usually, the alloy used in the deposition flux in the experiments is Al_0.33_Ga_0.67_As, i.e., *x_A_* = 0.33 in Equation 7.

**Figure 8:**
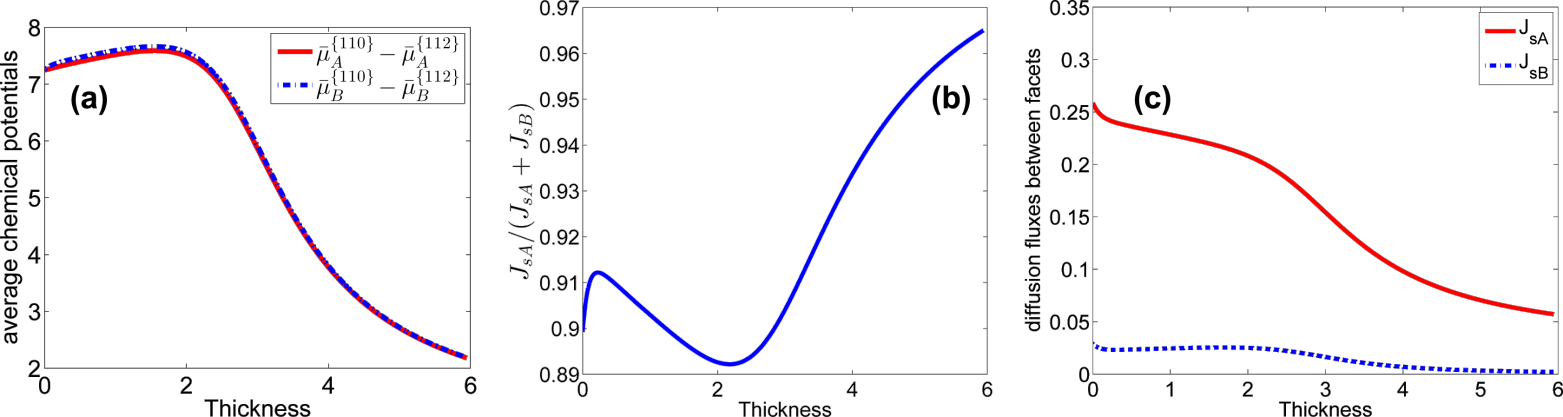
(a) Evolution of the difference of the average chemical potentials of the facets with the thickness of the shell(× 2.5 nm). (b) Evolution of the portion of Al atoms taken in the diffusion flux along the surface. (c) Evolution of the diffusion fluxes of Al atoms (denoted by A) and Ga atoms (denoted by B) from {110} facets to {112} facets.

**Figure 9:**
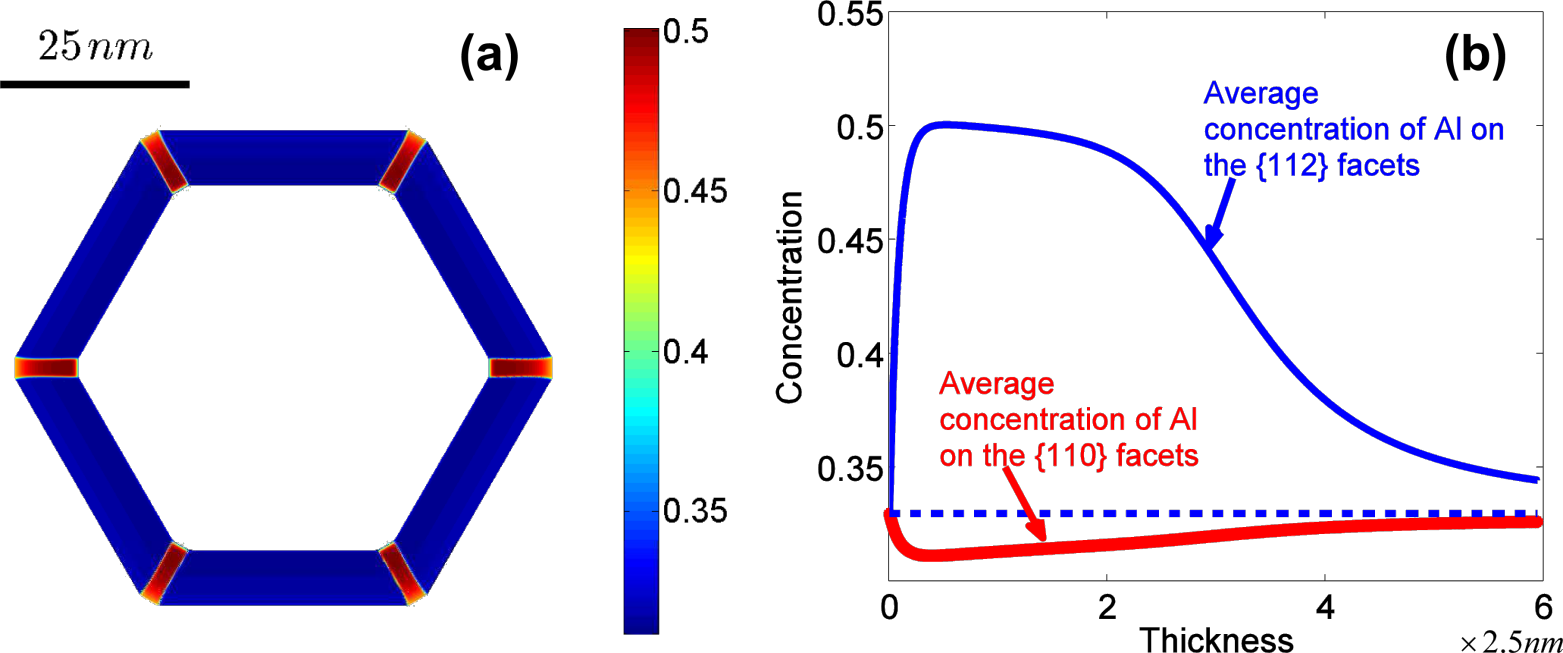
(a) Distribution of the concentration of Al in the shell of the nanowire. (b) Evolution of the average concentration of Al on different facets versus the thickness of the shell (in the direction normal to {110} facets).

In the numerical simulation, suppose the Al atoms diffuse much faster than Ga atoms on the {112} facets. For example, 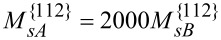. This is exaggerated, but it serves to illustrate the basic concepts that lead to stripes and dots. Under these conditions, it is shown first that both Al and Ga atoms tend to move from {110} to {112} facets because 
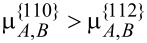
 (Figure 8a). Second, Figure 8b shows that the portion of Al atoms subjected to the tangential diffusion fluxes from {112} to {110} facets along the surface at the corners of {112} and {110} facets (*J_sA_*/(*J_sA_* + *J_sB_*)) are much larger than the portion of Al atoms subjected to the deposition flux that introduces the accumulation of Al atoms along the {112} facets (Figure 9). Moreover, this effect is enhanced within a certain thickness of the shell because surface diffusion dominates over deposition at early times. As time proceeds, the thickness of the shell of the nanowire gets large, diffusion gets weaker with a decrease of 
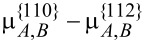
. Thus, *J_sA_* and *J_sB_* decrease (Figure 8a,c). The dynamic growth process switches from diffusion-dominated to deposition-dominated. This is why the concentration of Al atoms along the surface tends to be a constant equal to the concentration of Al atoms in the deposition flux when the thickness of the shell is large enough (Figure 9b).

**Figure 10:**
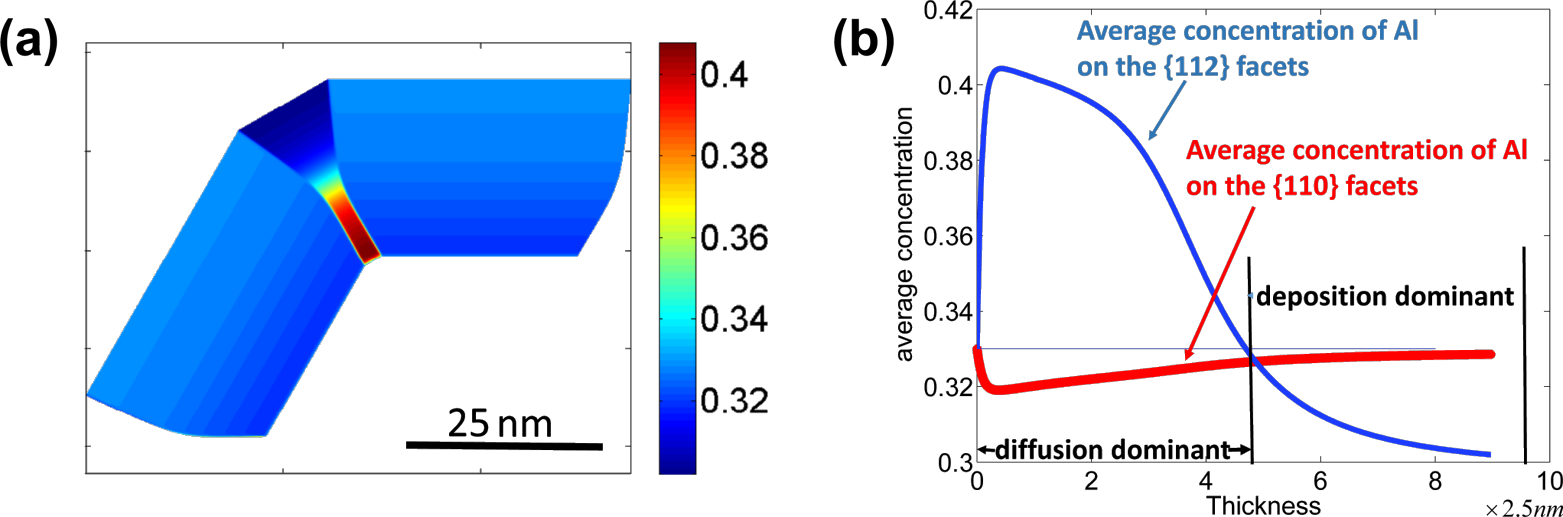
(a) Distribution of the concentration of Al in the shell of the nanowire. (b) Evolution of the average concentration of Al on different facets with the thickness of the shell (in the direction normal to {110} facets).

If it is harder for Al atoms to attach on the {112} than on the {110} facets from the deposition flux (for example, 
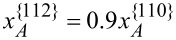
 and 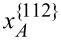 = 0.297, 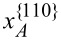 = 0.330) then the concentration of Al atoms along each facet tends to be a constant that is equal to its attachment rate on the corresponding facets in the deposition-dominated regime (Figure 10). The behaviour of the segregation is similar to the results of the former numerical simulation in the surface-diffusion-dominated regime (in this case 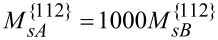). This explains why the Al-poor quantum dots can grow adjacent to an Al-rich stripe in some experiments [2].

**Figure 11:**
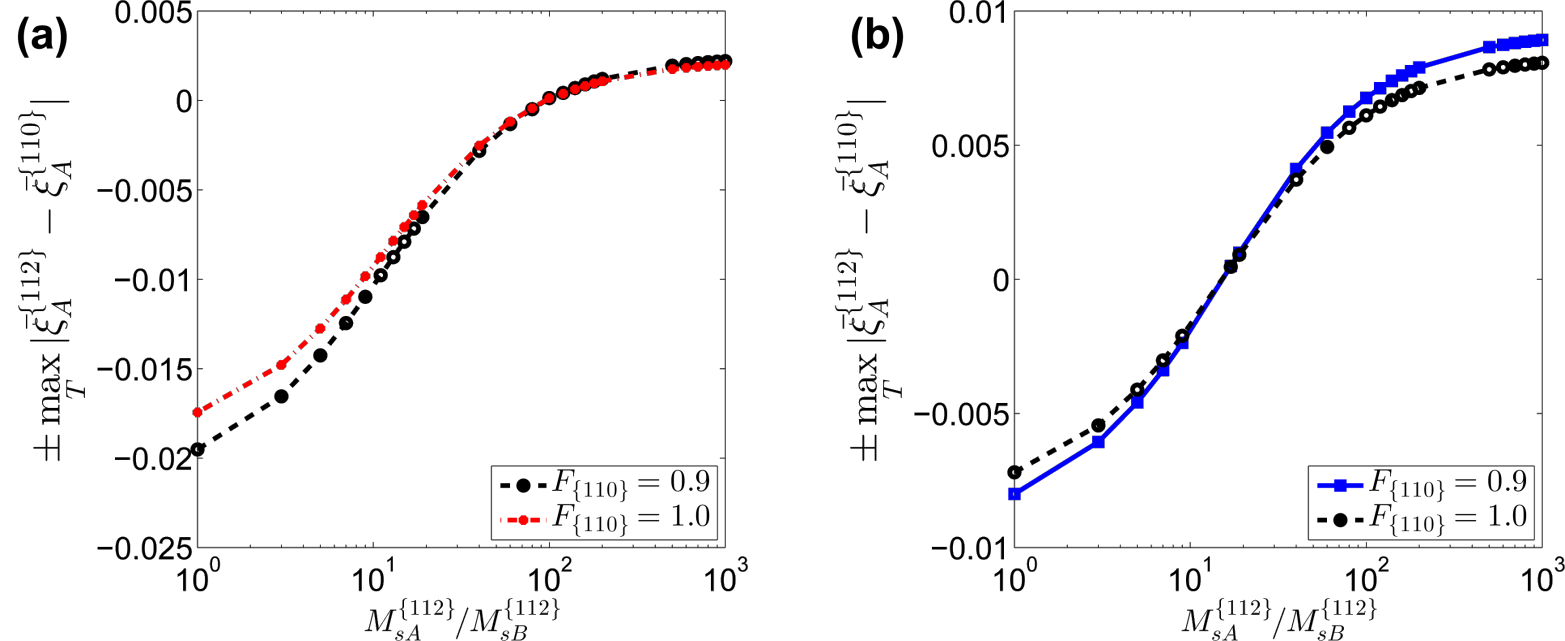
Influence of the ratio between the mobilities of different atoms and deposition rates on the surface concentration segregation. “+” means 
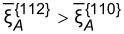
 whereas “−” means 
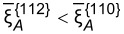
.

The presence of stripes and dots also strongly depends on the diffusivities of Al and Ga on the {110} facets. As the ratio between the diffusion coefficients on {110} facets approach unity, the ratio between the diffusion coefficients of Al between {110} and {112} facets necessary to get stripes and dots becomes much smaller than 1000. In particular, according to the results shown in Figure 11, a change from 
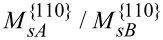
 = 0.1 to 
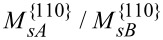
 = 0.5 will lead to a decrease of the critical value of 
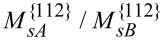
 to yield Al-rich stripes along the 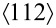 directions down to about a tenth.

It is worth mentioning that even though the morphological evolution is coupled with the mass transfer, as shown in Equation 7 and Equation 8, the surface segregation does not influence the morphological evolution significantly. This is because γ_0_ ≈ 1 J/m^2^ and *k*_B_Γ_0_*T* ≈ 0.01 J/m^2^. Hence, 
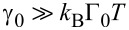
, which implies that 
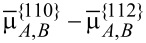
 is essentially determined by the surface energy densities of the pure materials on the facets. Therefore, the mechanisms for the morphological evolution in pure materials [7] are still applicable in the case of alloys.

## Acknowledgements

The authors would like to thank Jean-Noël Aqua, Lincoln J. Lauhon and Anna Fontcuberta i Morral for helpful discussions. This research was supported by the Office of Naval Research under Grant no. N00014-14-1-0697.
